# The Influence of the Flow Direction of KOH Solutions on the Measurement of Dissolved Hydrogen Permeability Through Alkaline Water Electrolysis Membranes

**DOI:** 10.3390/polym18081006

**Published:** 2026-04-21

**Authors:** Jun Hyun Lim, Jin Pyo Hwang, Euntaek Oh, Jinho Joo, Jian Hou, Chang Hyun Lee

**Affiliations:** Department of Energy Engineering, Dankook University, Cheonan 31116, Republic of Korea; iyj2368@naver.com (J.H.L.); hagollich@gmail.com (J.P.H.); bangasara1@naver.com (E.O.); id4765@naver.com (J.J.); houjimmy@naver.com (J.H.)

**Keywords:** alkaline water electrolysis, dissolved hydrogen permeability dead-end flow, cross-flow, concentration polarization

## Abstract

Alkaline water electrolysis (AWE) is a pivotal technology for sustainable hydrogen production. However, hydrogen permeation through its membranes remains a critical concern, as excessive gas crossover can lead to the formation of explosive mixtures and pose severe safety hazards. While conventional measurement techniques, such as pressure drop and electrochemical methods, are suitable for porous membranes, they exhibit inherent limitations when applied to dense membranes such as anion exchange membranes. This study proposes a cross-flow measurement methodology applicable to all types of AWE membranes. Unlike traditional dead-end configurations, the cross-flow approach effectively mitigates impurity accumulation and maintains a continuous electrolyte flow parallel to the membrane surface. This configuration ensures uniform electrolyte distribution, minimizes local concentration and pressure fluctuations, and enhances measurement reliability and reproducibility relative to the conventional dead-end flow. Furthermore, the methodology ensures accurate and reproducible measurements, demonstrating enhanced detection capability for dense membranes with intrinsically low permeability by mitigating fouling and concentration polarization effects. These findings provide a robust framework for the development of high-performance membranes designed to suppress dissolved hydrogen permeability.

## 1. Introduction

Hydrogen is increasingly recognized as a pivotal energy carrier in the transition toward a carbon-neutral society [1,2]. Among various hydrogen production technologies, alkaline water electrolysis (AWE) is considered one of the most promising due to its economic viability and scalability [3,4,5,6,7]. One of the critical factors influencing the efficiency and safety of AWE systems is the permeability of dissolved hydrogen through the membrane separating the anode and cathode compartments [8,9]. Failure to adequately control hydrogen permeation can result in gas crossover and the formation of explosive gas mixtures, thereby undermining system performance and safety [10,11,12,13].

Previous studies have primarily focused on porous membranes, employing techniques such as pressure decay and electrochemical methods to measure hydrogen permeability [14,15]. However, the recent integration of dense membranes into AWE systems has presented new challenges. These membranes exhibit significantly lower permeability, rendering conventional methods inadequate for accurate assessment [16,17]. Moreover, many existing techniques are poorly suited to the highly alkaline operating conditions of AWE (e.g., 30 wt.% KOH at 80 °C), leading to unreliable data [9,18]. Notably, no previous study has systematically adapted cross-flow hydrodynamics to such extreme alkaline electrolysis environments. The key novelty of our work lies in overcoming these multi-faceted technical challenges to establish a reliable measurement platform capable of quantifying dissolved hydrogen permeability under realistic electrolysis conditions.

A widely used method is the dead-end flow technique, where pressure is applied perpendicularly to the membrane surface to drive permeation [19,20]. However, this approach often suffers from impurity accumulation and concentration polarization on the membrane surface during long-term testing, resulting in the underestimation of actual permeability [21,22,23]. These issues are particularly pronounced in dense membranes, where the discrepancy between measured and actual values becomes more significant [17,24].

In contrast, practical AWE systems operate under cross-flow configurations, where the electrolyte flows horizontally along the membrane surface [25,26]. This structural design effectively mitigates membrane fouling, maintains uniform pressure and concentration distribution, and thereby establishes a more stable and reproducible measurement environment [17,27]. Consequently, the development of a cross-flow-based measurement system that mimics the operational conditions of AWE is essential for accurately evaluating the permeability of dissolved hydrogen in dense membranes.

**Figure 1:**
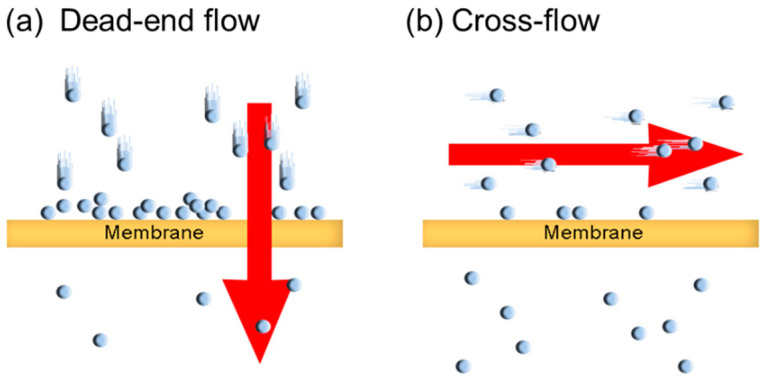
Schematic of the dead-end flow and cross-flow systems.

To visually illustrate the difference between conventional dead-end flow and the proposed cross-flow configuration, Figure 1 presents a schematic diagram of both systems. In this study, we propose a novel cross-flow measurement system designed to simulate the actual operating conditions of AWE systems. Using this customized system, we systematically investigate the permeation behavior of diverse membranes and compare the results with those obtained via conventional dead-end flow. Our findings demonstrate significant advantages of the cross-flow approach, including enhanced sensitivity, reduced membrane contamination, and minimized concentration polarization effects. Thus, the primary aim of this study is to develop and validate a robust cross-flow measurement system for quantifying dissolved hydrogen permeability under industrially relevant alkaline conditions. This work highlights the methodological novelty of our approach, which transcends simple membrane-to-membrane comparisons. These results provide valuable insights for the development of high-performance membranes, thereby improving the safety and efficiency of AWE systems.

## 2. Experimental

### 2.1. Materials

The reference materials used in this study were Agfa’s porous separator Zirfon^®^, which is widely used commercially in AWE, and Dioxide Materials’ Sustainion^®^ as an anion exchange membrane [5,28,29]. The perfluorinated ionomer used to synthesize the perfluorinated anion exchange ionomer was a perfluorinated ionomer (PFI) precursor with ionomer equivalent weights (EWs) of 725 and 1100, respectively. The reagents 1-propanol (NPA, purity ≥ 99.9%), N-phenylhydroxylamine (purity = 97.0%), and 1-Methyl-2-Pyrrolidone (NMP, purity = 99.5%) were purchased from Sigma-Aldrich (Burlington, MA, USA). Potassium hydroxide (KOH, purity > 93%) was purchased from Daejung Chemical (Siheung-si, Republic of Korea). All chemical reagents were used as received without additional purification.

### 2.2. Ionomer Synthesis and Membrane Fabrication

The perfluorinated (PF) ionomers synthesized in this study correspond to the NR-HPA and 3M-HPA membranes used in subsequent experiments. For synthesis, the perfluorinated anion exchange ionomer was prepared by mixing a PF-SO_2_F precursor and N-phenylhydroxylamine in a reactor containing an excess 0.5 M NaOH solution at a controlled ratio. The reaction mixture was heated to 150 °C and stirred under reflux for 24 h. After cooling to 25 °C, the resulting precipitated powder was filtered, repeatedly washed with deionized water, and dried at 60 °C for one day to obtain a powder-type ionomer (PF-HPA) [30]. To fabricate membranes, PF-HPA nano-dispersions prepared via a supercritical method were subjected to ultrasonic treatment to remove microbubbles. The dispersions were then cast using a solution-casting method, followed by sequential thermal processing: drying at 60 °C for 6 h, heating at 85 °C for 6 h, and annealing at 220 °C for 12 min under vacuum. These procedures yielded dense membranes with controlled thickness, which were designated as NR-HPA and 3M-HPA in this study [30]. The fundamental characteristics of the membranes employed in this study, including morphology type and thickness, are summarized in Table 1.

### 2.3. Dissolved Hydrogen Permeability Using the Dead-End Flow Method

**Figure 2:**
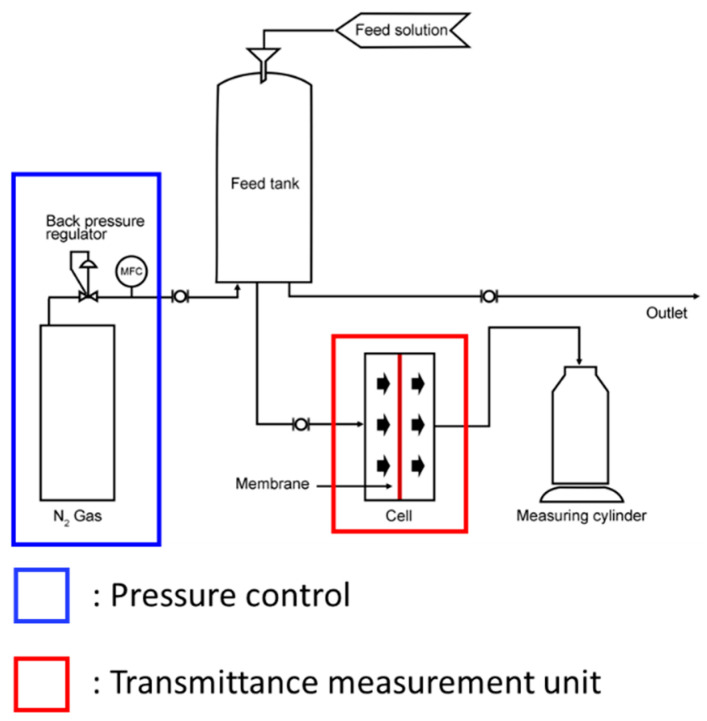
Schematic of dissolved hydrogen permeability measurement equipment using the dead-end flow method.

After loading the measurement membrane inside the nickel cell, it was secured under a specific pressure. The effective area of the nickel cell is 7 × 7 cm^2^. Completely fill the supply tank with 30 wt.% KOH electrolyte solution. Set the temperature condition to 80 °C using a heating jacket and stabilize it. After connecting to the fastened cell, apply absolute pressure in the range of 1.1 to 1.5 bar using a mass flow meter and a back pressure regulator. Then measure the mass of the 30 wt.% KOH aqueous solution being transmitted. The schematic of the dissolved hydrogen permeability measurement equipment using the dead-end flow method is shown in Figure 2.

According to Darcy’s law, the dissolved hydrogen permeability $\varepsilon_{H_{2}}^{Darcy}$ can be determined using Equation (1) [14,15]:

(1) $$\varepsilon_{H_{2}}^{Darcy}=\;-d\frac{d(\Phi_{el})}{d(∆P)}S_{H_{2}}P_{H_{2}}^{Cat}$$

Here, $\varepsilon_{H_{2}}^{Darcy}$ is the dissolved hydrogen permeability driven by the pressure difference (mol s^−1^ cm^−1^ bar^−1^), *d* denotes the characteristic thickness of the electrolyte membrane (cm), $\Phi_{el}$ is the volumetric electrolyte flux normalized to the cell area (cm^3^ cm^−2^ s^−1^ bar^−1^), ∆P is the absolute pressure difference between both sides of the membrane (bar), $S_{H_{2}}$ is the hydrogen solubility (mol cm^−3^ bar^−1^), and $P_{H_{2}}^{Cat}$ is the applied pressure (bar).

### 2.4. Dissolved Hydrogen Permeability Using the Developed Cross-Flow Method

**Figure 3:**
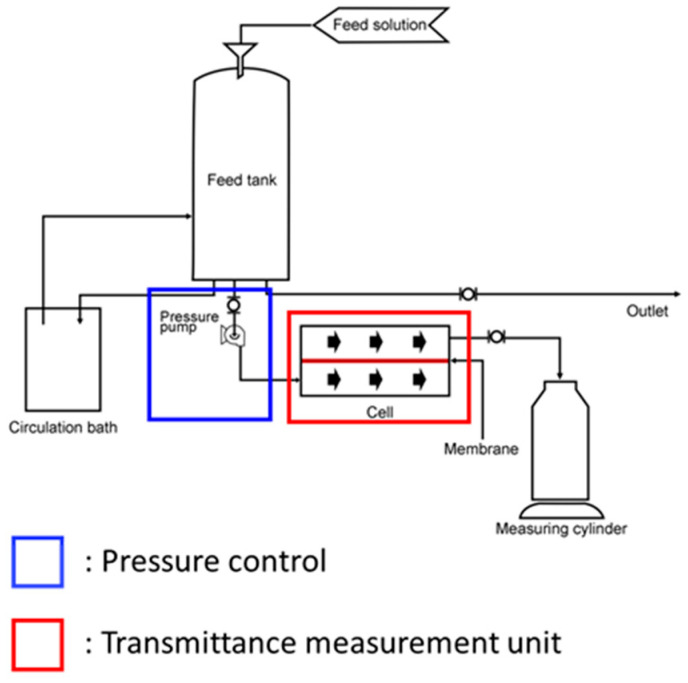
Schematic of dissolved hydrogen permeability measurement equipment developed using the cross-flow method.

Dissolved hydrogen permeability analysis was performed to quantify the dissolved hydrogen flux through the membrane separating the anode and cathode in an AWE system. The analysis utilized a 30 wt.% KOH electrolyte solution, maintained at 80 °C to replicate operational conditions of the AWE system. Membranes (active area, 5 cm × 2 cm) were used to fit the size of the double O-ring gasket installed in the module to prevent leakage of the alkaline solution under high pressure. The system was initially stabilized by circulating the feed solution. Subsequently, the feed solution flow rate was incrementally increased by adjusting the RPM of the supply pump, and the system was sequentially pressurized to the permeation onset pressure range (e.g., 0.1 to 0.5 bar for a porous membrane and 0 to 20 bar for an anion exchange membrane). The mass of the permeated solution was recorded over time from the point of permeation onset. The dissolved hydrogen permeability was then calculated according to Darcy’s law, using the previously defined Equation (1). Figure 3 shows the schematic of the dissolved hydrogen permeability measurement equipment developed using the cross-flow method.

### 2.5. Surface and Cross-Sectional Analysis (SEM)

To investigate the surface and cross-sectional morphology of the membranes, a field-emission scanning electron microscope (FE-SEM, HITACHI S-4700, Tokyo, Japan) was employed. Samples included pristine membranes as well as those subjected to dead-end and cross-flow permeability measurements, allowing for comparative analysis of structural changes under different testing conditions. SEM images were obtained at magnifications of ×100 and ×10,000, with particular emphasis placed on identifying surface contamination and observing morphological changes. In addition, energy-dispersive X-ray spectroscopy (EDS) analysis was performed using an integrated detector system to investigate the elemental composition of surface-bound deposits.

## 3. Results and Discussion

### 3.1. Comparison of Dissolved Hydrogen Permeability in Zirfon^®^ Membranes Using the Dead-End Flow and Cross-Flow Methods

**Figure 4:**
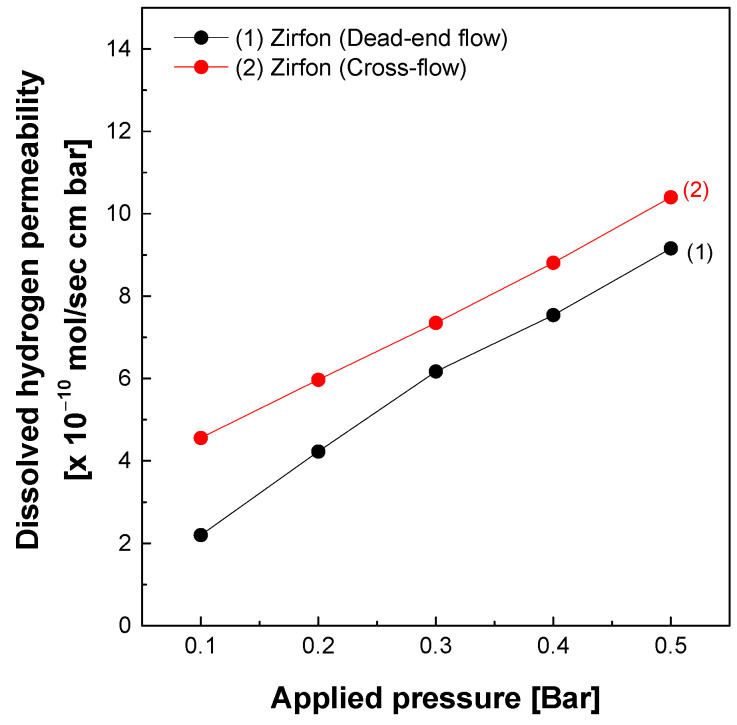
Dissolved hydrogen permeability analysis of Zirfon^®^ according to the dead-end flow and cross-flow methods.

The dissolved hydrogen permeability of Zirfon^®^ membranes was evaluated using both dead-end and cross-flow measurement methods. As shown in Figure 4, permeability increased with applied pressure in both methods, indicating that permeation flux is pressure-dependent, which is consistent with Darcy’s law [22,31]. Notably, at identical pressure conditions, the cross-flow method consistently exhibited higher permeability than the dead-end flow.

For example, at an applied pressure of 0.3 bar, the permeability measured in the dead-end flow was approximately 6.17 × 10^−10^ mol·s^−1^·cm^−1^·bar^−1^, whereas the cross-flow method measured a higher value of approximately 7.35 × 10^−10^ mol·s^−1^·cm^−1^·bar^−1^. This discrepancy is attributed to differences in mass transport resistance caused by concentration polarization and surface contamination. In the dead-end flow, stagnant electrolyte near the membrane surface promotes the accumulation of OH^−^ and K^+^ ions, leading to the formation of KOH precipitates. These deposits hinder hydrogen dissolution and diffusion pathways, resulting in an increased concentration gradient across the membrane interface and a gradual decline in permeability slope [23,32]. Although the present study did not attempt to quantify the polarization layer thickness, established models such as film theory describe concentration polarization as the formation of a stagnant boundary layer that increases mass transport resistance.

Indeed, the permeability curve obtained from the dead-end flow exhibited a nonlinear trend. The slope decreased at higher pressures, indicating increased transport resistance resulting from concentration polarization. In contrast, the cross-flow method maintained a continuous electrolyte flow across the membrane surface, effectively suppressing the accumulation of contaminants and promoting uniform pressure and concentration distribution. As a result, the permeability response remained more linear with increasing pressure, reflecting stable and consistent transport conditions throughout the measurement. It should be noted that the higher permeability observed in the cross-flow configuration does not indicate instability of the membrane, but rather reflects the suppression of artifacts such as concentration polarization and surface fouling that lead to underestimation in the dead-end flow. Thus, the cross-flow values represent the intrinsic permeability of the membrane under stable operating conditions.

### 3.2. Time-Dependent Changes in Dissolved Hydrogen Permeability According to Measurement Method

As previously discussed, the dead-end flow inherently promotes electrolyte stagnation near the membrane surface, leading to the accumulation of KOH precipitates and the onset of concentration polarization [22,23,33]. These phenomena directly affect the long-term stability of hydrogen permeability and limit the ability of this method to accurately simulate real operating conditions in electrochemical systems.

**Figure 5:**
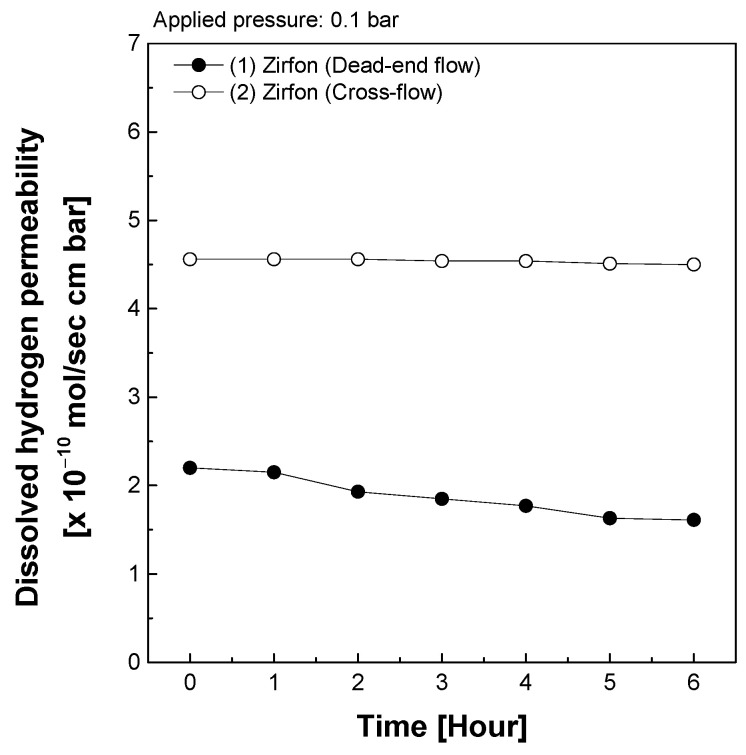
Changes in dissolved hydrogen permeability of Zirfon^®^ over time.

To evaluate this effect, the dissolved hydrogen permeability of Zirfon^®^ membranes was measured continuously for 6 h with measurements taken every hour, while maintaining a constant applied pressure of 0.1 bar. As shown in Figure 5, the cross-flow configuration maintained a stable permeability of approximately 4.5 × 10^−10^ mol·s^−1^·cm^−1^·bar^−1^ throughout the entire duration. In contrast, the dead-end configuration exhibited an initial permeability of approximately 2.2 × 10^−10^ mol·s^−1^·cm^−1^·bar^−1^, which gradually declined to approximately 1.6 × 10^−10^ mol·s^−1^·cm^−1^·bar^−1^ after 6 h [5,14].

This reduction in permeability is attributed to increased transport resistance caused by concentration polarization and surface contamination. In the dead-end flow, KOH salts accumulate on the membrane surface and form crystalline deposits, progressively obstructing hydrogen dissolution and diffusion pathways. As a result, the concentration gradient across the membrane–electrolyte interface intensifies over time, leading to a steady decline in permeability. In contrast, the cross-flow method enables continuous electrolyte flow along the membrane surface, effectively suppressing contaminant buildup and maintaining uniform pressure and concentration distribution across the membrane. A detailed theoretical analysis of the polarization layer thickness and its impact on hydrogen transport is beyond the scope of this study. We acknowledge this as a limitation and suggest that future work should focus on modeling the polarization layer and its quantitative influence on hydrogen permeability. Consequently, concentration polarization does not occur, and the permeability remains stable even under prolonged measurement conditions.

These findings demonstrate that the cross-flow method offers superior measurement stability and reproducibility, not only in short-term evaluations but also under extended operating conditions. This supports its validity as a reliable method for simulating real-world environments in AWE systems. The apparent increase in permeability compared to the dead-end flow is therefore consistent with improved measurement accuracy, as the cross-flow system prevents contaminant buildup and reveals the true transport properties of the membrane while maintaining long-term stability.

### 3.3. Structural Analysis of Zirfon^®^ Membranes Before and After Permeability Testing (FE-SEM)

To investigate the structural characteristics of the Zirfon^®^ membrane, field-emission scanning electron microscopy (FE-SEM) was employed to observe both surface and cross-sectional morphologies. The analysis focused on changes in pore structure and surface contamination before and after hydrogen permeability testing.

**Figure 6:**
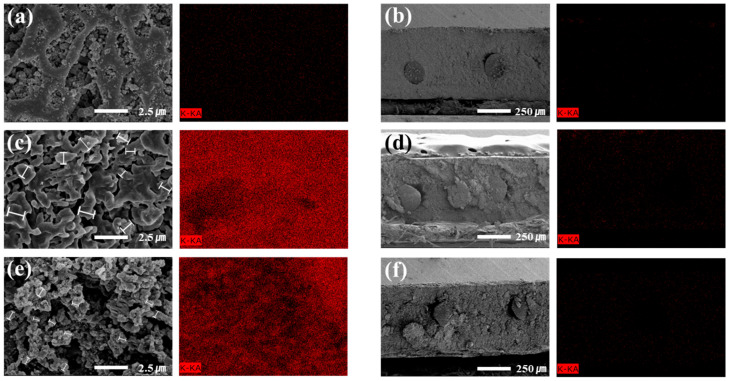
Surface and cross-sectional SEM images of Zirfon^®^ membranes before and after permeability testing: (**a**) pristine surface, (**b**) pristine cross-section, (**c**) surface after dead-end flow test, (**d**) cross-section after dead-end flow test, (**e**) surface after cross-flow test, and (**f**) cross-section after cross-flow test.

As shown in Figure 6a,b, the pristine membrane exhibited a clean surface with a well-defined porous structure and maintained a uniform thickness across the cross-section. These features provide a structural foundation for high permeability and stable physical performance.

In contrast, the membrane tested under dead-end flow conditions (Figure 6c,d) showed significant surface contamination, with fine particulate deposits partially blocking the pores. Surface observations confirmed that the average particle size of the precipitates was greater than 1 µm. Cross-sectional observations revealed the accumulation of KOH precipitates near the surface, forming a thin layer that could interfere with hydrogen dissolution and diffusion. These structural changes are closely associated with the concentration polarization and increased transport resistance discussed earlier, and are considered contributing factors to the observed decline in permeability during long-term measurements.

On the other hand, the membrane tested under cross-flow conditions (Figure 6e,f) exhibited noticeably fewer surface deposits compared to the dead-end sample. The average particle size of the precipitates was approximately 0.6 µm, which is about 40% smaller than that observed in the dead-end measurement samples. The cross-sectional morphology remained uniform in both thickness and structure, with no visible signs of precipitate accumulation or physical damage. This suggests that continuous electrolyte flow along the membrane surface effectively suppresses contaminant buildup and maintains uniform pressure and concentration distribution across the membrane.

To complement the morphological observations, EDS analysis was conducted to evaluate the elemental composition of surface-bound precipitates on the membrane. Table 2 summarizes the EDS mapping results by elemental species. Particular focus was placed on mapping the distribution of potassium (K), a representative component of the alkaline electrolyte (KOH), to confirm the presence of residual KOH on the membrane surface. As shown in Table 2, the quantitative EDS data clearly demonstrate the differences in potassium deposition between dead-end and cross-flow conditions, thereby supporting the discussion of fouling and concentration polarization.

Based on the EDS mapping results, no significant K signals were detected on either the surface or cross-section of the pristine membrane, with values as low as 0.01 wt.% and 0.16 wt.%, respectively. This confirms the absence of KOH-derived residues in the initial membrane state and supports its role as a reference baseline.

In contrast, the membrane tested under dead-end flow conditions exhibited substantial potassium accumulation. The surface showed a high K content of 46.17 wt.%, while the cross-section contained 4.56 wt.%. These results indicate extensive deposition of KOH-derived precipitates, likely caused by stagnant electrolyte conditions. The elevated potassium levels suggest that such deposits may physically obstruct hydrogen dissolution and diffusion pathways. Additionally, the presence of K within the cross-section implies partial penetration of the precipitates into the membrane structure.

For the membrane tested under cross-flow conditions, potassium signals were significantly reduced. The surface contained 19.85 wt.% K, and the cross-section showed 3.36 wt.%, representing approximately 57% and 26% of the values observed in the dead-end sample, respectively. This reduction demonstrates that continuous electrolyte flow effectively suppresses precipitate formation and minimizes surface contamination. SEM observations further confirmed that the cross-flow sample maintained a cleaner surface and more stable morphology.

Overall, the SEM and EDS analyses visually demonstrate that the structural integrity and surface contamination of the membrane are significantly influenced by the flow method. The cross-flow method provides a more favorable environment for preserving the physical integrity of the membrane and ensuring long-term operational stability. These findings support the validity of the cross-flow approach as a reliable measurement method for performance evaluation in AWE systems.

### 3.4. Evaluation of Measurement Precision and Applicability of the Cross-Flow Method

**Figure 7:**
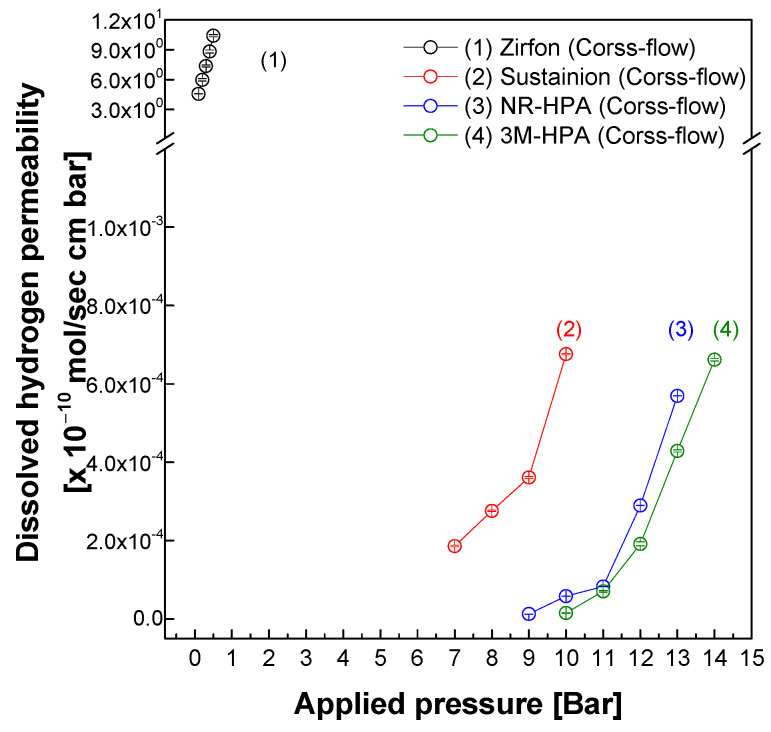
Dissolved hydrogen permeability of the membranes via cross-flow.

Figure 7 presents the results of dissolved hydrogen permeability measurements conducted under cross-flow conditions using various membrane materials, including Zirfon^®^, Sustainion^®^, NR-HPA, and 3M-HPA. Zirfon^®^ is characterized by a porous structure, whereas Sustainion^®^ and the HPA-based membranes exhibit dense morphologies. Despite these structural differences, all membranes showed an increasing trend in permeability with rising pressure, indicating that the cross-flow method enables stable and precise measurements across membranes with diverse structural properties. While the cross-flow system enables the detection of permeability values significantly lower than those measurable by the dead-end flow, the absolute limit of detection (LOD) for dissolved hydrogen flux was not determined in this study. Reproducibility at very low concentrations was qualitatively confirmed via stable repeated measurements; however, quantitative metrological validation (e.g., establishing the minimum detectable flow rate or conducting statistical error analysis) remains a subject for future work.

Due to its porous architecture, Zirfon^®^ exhibited relatively high permeability even at low pressures (0–0.5 bar), maintaining a linear increase as pressure rose. In contrast, the dense membranes demonstrated noticeable permeability behavior only at elevated pressures: above 7 bar for Sustainion^®^, 9 bar for NR-HPA, and 10 bar for 3M-HPA. Notably, although the absolute permeability values of dense membranes were approximately 10,000 times lower than those of porous membranes, the cross-flow setup provided a highly sensitive measurement environment capable of detecting subtle changes in permeability. This allowed for reliable data acquisition even in membranes with high transport resistance, facilitating accurate performance comparison and quantitative evaluation.

In conclusion, the cross-flow method is broadly applicable to a wide range of membrane types regardless of their structural characteristics. It serves as a robust analytical tool, particularly effective for high-resolution measurements in dense membranes. This approach offers a valuable experimental foundation for assessing and optimizing membrane performance in practical applications such as water electrolysis and gas separation processes.

## 4. Conclusions

This study successfully showed the development of a cross-flow methodology for measuring dissolved hydrogen permeability under strongly alkaline conditions, marking a significant advancement in the AWE field. This innovative approach effectively addresses the fundamental limitations of conventional dead-end flow, particularly when characterizing non-porous membranes. By facilitating a continuous electrolyte flow parallel to the membrane surface, the cross-flow configuration mitigates impurity accumulation and maintains a stable, uniform test environment. This uniformity minimizes local fluctuations in concentration and pressure—factors that typically distort permeability measurements—thereby ensuring superior reliability and reproducibility compared to the dead-end flow, while maintaining consistency with values reported in the literature.

Experimental validation demonstrates that the proposed method exhibits enhanced sensitivity, making it exceptionally effective for evaluating AWE membranes with low hydrogen transport rates. These findings not only deepen the fundamental understanding of hydrogen permeation phenomena but also provide critical empirical data to guide the design of high-performance membranes. By enabling precise evaluation under conditions consistent with actual system operations, this methodology supports the development of materials optimized for safety and efficiency in practical hydrogen production applications. Ultimately, the implementation of this cross-flow approach will contribute to the mitigation of hydrogen crossover risk, enhancing the overall viability and safety of sustainable AWE systems.

## Figures and Tables

**Table 1:** Fundamental characteristics of membranes for AWE.

Sample	Morphology Type	Thickness [μm]
Zirfon^®^	Porous	500 ± 30
Sustainion^®^	Dense	50 ± 5
NR-HPA	Dense	50 ± 2
3M-HPA	Dense	50 ± 2

**Table 2:** Elemental composition of Zirfon^®^ membrane surfaces and cross-sections under different flow conditions.

	(a) Surface before test			(b) Cross-section before test		
	Weight [%]	Atomic [%]	Error [wt.%]	Weight [%]	Atomic [%]	Error [wt.%]
K	0.01	0.01	0.08	0.16	0.08	0.11
Zr	48.11	11.81	5.46	32.78	6.87	3.51
C	35.97	67.05	12.97	44.56	70.91	19.38
O	14.31	20.02	5.44	14.58	17.42	7.35
S	1.60	1.12	0.25	7.93	4.73	0.89
Total	100.00	100.00	-	100.00	100.00	-
	(c) Surface after dead-end flow test			(d) Cross-section after dead-end flow test		
	Weight [%]	Atomic [%]	Error [wt.%]	Weight [%]	Atomic [%]	Error [wt.%]
K	46.17	25.18	4.03	4.56	2.39	0.51
Zr	3.37	0.79	0.44	36.30	8.16	3.98
C	15.45	27.43	5.51	35.82	61.16	16.22
O	34.96	46.58	12.08	20.82	26.69	9.73
S	0.04	0.03	0.08	2.50	1.60	0.37
Total	100.00	100.00	-	100.00	100.00	-
	(e) Surface after cross-flow test			(f) Cross-section after cross-flow test		
	Weight [%]	Atomic [%]	Error [wt.%]	Weight [%]	Atomic [%]	Error [wt.%]
K	19.85	10.14	1.87	3.36	1.76	0.43
Zr	21.97	4.81	2.57	37.85	8.49	4.37
C	31.38	52.19	11.34	38.47	65.51	18.81
O	25.85	32.27	9.44	17.60	22.50	9.38
S	0.95	0.59	0.18	2.73	1.74	0.42
Total	100.00	100.00	-	100.00	100.00	-

## Data Availability

The original contributions presented in this study are included in the article. Further inquiries can be directed to the corresponding author.
